# Morphometric features enhance phenotype discrimination in frontotemporal lobar degeneration

**DOI:** 10.1093/braincomms/fcag012

**Published:** 2026-02-02

**Authors:** Jane K Stocks, Ashley A Heywood, Karteek Popuri, Mirza Faisal Beg, Howard J Rosen, Lei Wang

**Affiliations:** Department of Psychiatry and Behavioral Sciences, Feinberg School of Medicine, Northwestern University, Chicago, IL 60611, USA; Department of Psychiatry and Behavioral Sciences, Feinberg School of Medicine, Northwestern University, Chicago, IL 60611, USA; Department of Computer Science, Memorial University of Newfoundland, St. John's, NL, Canada A1C5S7; School of Engineering Science, Simon Fraser University, Burnaby, BC, Canada V5A1S6; School of Medicine, University of California, San Francisco 94143, USA; Department of Psychiatry and Behavioral Sciences, Feinberg School of Medicine, Northwestern University, Chicago, IL 60611, USA; Department of Psychiatry and Behavioral Health, Ohio State University Wexner Medical Center, Columbus, OH 43210, USA

**Keywords:** morphometry, MRI, frontotemporal lobar degeneration, frontotemporal dementia, phenotype discrimination

## Abstract

Frontotemporal lobar degeneration is associated with diverse clinical phenotypes underlain by multiple disease pathologies and genetic mutations for which traditional structural magnetic resonance imaging (MRI) analyses lack discriminatory sensitivity and specificity. Here, we use a data-driven multivariate method to extract a concise set of MRI-derived shape morphometric features and cross-sectionally examine the discriminatory capability of their unique combinations in three frontotemporal lobar degeneration clinical phenotypes. Patients with sporadic or familial frontotemporal lobar degeneration clinical syndromes across two cohorts (i.e. behavioral variant (*n* = 173), non-fluent variant primary progressive aphasia (*n* = 63), semantic variant primary progressive aphasia (*n* = 41)) and 158 controls were assessed. Cortical morphometry measures of cortical thickness, surface curvature, and metric distortion were extracted, contrasted with controls using linear models, and additionally entered into a sparse partial least squares discriminatory analysis (sPLS-DA) designed to model multimodal signatures unique to each phenotype. Discriminatory power of partial least squares-derived features was tested on independent, age-matched test data. We found that each cortical morphometric feature significantly differed between clinical syndromes in dissociable spatial patterns. On independent data, the combination of cortical thickness and surface curvature best discriminated between behavioural variant and non-fluent variant primary progressive aphasia patients from controls. For semantic variant primary progressive aphasia, any model including cortical thickness maximized model performance. The sparse partial least squares approach indicated distinctive brain regions contribute to discrimination for each shape feature, suggesting each feature may reflect unique aspects of neurodegeneration across groups. This method could prove invaluable in future studies for early detection of frontotemporal lobar degeneration phenotypes.

## Teaser

Stocks *et al*. report that integrating several magnetic resonance imaging-based measures of cortical shape improves the ability to distinguish between clinical forms of frontotemporal lobar degeneration. Their findings demonstrate that combining cortical thickness, curvature, and surface distortion provides more accurate and biologically specific markers of disease than any single measure alone.

## Graphical Abstract

**Graphical Abstract:**
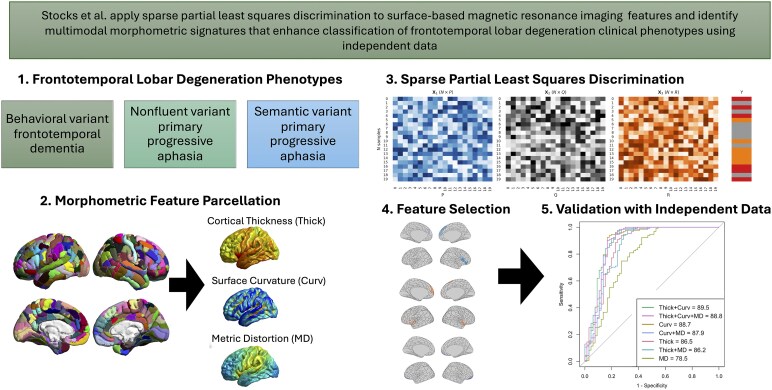

## Introduction

Frontotemporal lobar degeneration (FTLD) is a group of heterogeneous neurodegenerative diseases marked by the accumulation of toxic protein aggregates in the central nervous system, usually containing either misfolding of tau (FTLD-tau), transactive response DNA binding protein 43 kDa (TDP-43), or fused in sarcoma (FUS) protein.^1^ FTLD is clinically heterogeneous, with its canonical syndromes characterized by specific constellations of progressive deficits in behaviour, language, and/or cognition.^2,3^ The most common clinical phenotypes associated with underlying FTLD neuropathology include behavioural variant frontotemporal dementia (bvFTD), primary progressive aphasia variants (semantic (svPPA), non-fluent/agrammatic, (nfvPPA) and logopenic (lvPPA)), corticobasal syndrome (CBS), progressive supranuclear palsy (PSP), and FTD with motor neuron disease (FTD-MND).^4^ Moreover, highly penetrant autosomal mutations account for up to a third of FTLD cases, usually due to mutations in the *MAPT*, *GRN,* or *C9orf72* genes.^5,6^

Although clinical trials of potential disease-altering therapies (e.g. anti-tau antibodies, tau aggregation inhibitors) are currently underway,^7-9^ the significant degree of clinical, pathological and genetic heterogeneity observed in FTLD hinders the development of sensitive and specific biomarkers that would allow for targeted recruitment of groups at highest risk for clinical/cognitive decline into clinical trials for intervention.^10^ As such, more recent work in FTLD biomarker development has begun to focus on earlier stages of illness,^11-13^ driven by data indicating that biological changes in other neurodegenerative conditions can begin up to ten to twenty years before symptom onset.^14-16^ When assessed using MRI, FTLD clinical syndromes are typically associated with distinguishable neuroanatomical patterns; the earliest neuroanatomical changes typically involve the insula and anterior cingulate in bvFTD,^17,18^ the anterior temporal lobes in svPPA,^19^ and the inferior frontal gyrus in nfvPPA.^20^ Interestingly, studies that have examined individuals with similar clinical phenotypes underlain by different gene mutations have typically found similar patterns of neurodegeneration, highlighting that the location of pathological accumulation results in common clinical presentations of disease, despite being caused by distinct genetic mutations (and consequently, sometimes also neuropathologies).^21^ Further, considerable similarities exist between grey and white matter abnormalities among familial and sporadic FTLD clinical syndromes.^22^ It is of utmost importance to develop biomarkers that are specific to FTLD clinical phenotypes, even across multiple underlying mutations or pathologies.

To that end, researchers have examined whether more sensitive measures of neurodegeneration can be derived from a single MRI scan, with the intention of improving sensitivity beyond volume-based studies of neurodegeneration. Surface-based approaches provide improved spatial localization and alignment of cortical regions compared to volume-based methods, thereby enhancing sensitivity to subtle cortical changes.^23-25^ These advantages make surface-derived measures particularly well-suited for studying neurodegeneration, where cortical abnormalities are often regionally specific. Examples of such measures include cortical thickness, sulcal depth, surface area, metric distortion and surface curvature. In studies of Alzheimer’s disease (AD) related neuropathological change, there is evidence that shape morphometric features such as cortical thinning,^26^ sulcal depth,^27^ and curvature^28^ improve classification of early disease-stage individuals versus controls. Moreover, shape morphometry measures may provide critical insight into the early pathogenic factors that contribute to neurodegeneration, as some features, such as gyrification, have been proposed to reflect abnormal neurodevelopment or early brain insults.^29^ In FTLD, cortical folding abnormalities have been reported in presymptomatic mutation carriers, such that *C9orf72* carriers showed significantly abnormal gyrification in the left frontal and right parieto-occipital regions, as early as 10–20 years before projected symptom onset.^30^

Despite this promising, highly sensitive method for early detection of disease, methods that aim to extract multiple morphometric features for analysis across groups of subjects must additionally grapple with the high-dimensionality and potentially correlated structure of information across features. Multi-modal integrative frameworks that additionally incorporate feature reduction, such as principal component analysis (PCA) or partial-least squares (PLS) regression, are optimally designed for such tasks.^31^ PLS methods have the ability to handle high-dimensional, multicollinear data, even among data sets even with low sample sizes, all while additionally searching for multimodal signatures that explain the maximum covariance between the explanatory and response variables.^32^ In contrast to traditional PLS, sparse PLS (sPLS) imposes a sparsity constraint that selects only the most informative variables for each component, thereby reducing overfitting and improving interpretability in high-dimensional neuroimaging data. To that end, several studies have shown that combining multiple imaging modalities via multivariate integration methods improves diagnostic classification accuracy in neurodegenerative conditions.^33-39^ In addition, prior work has demonstrated that volumetric MRI combined with machine learning can discriminate FTD from controls with high accuracy.^40^ More recently, Metz *et al*.^41^ combined deformation-based morphometry with cognitive features in participants with FTD syndromes, using partial least squares and machine learning to classify groups with high accuracy, underscoring the utility of multivariate methods for FTD. Our study extends these approaches by focusing on surface-based morphometric features (thickness, curvature, and metric distortion), applying sPLS for multimodal feature selection, and validating results in an independent dataset. It is important to note, however, that clinical syndromes do not map one-to-one onto underlying neuropathology or genetics, which are ultimately the most relevant targets for disease-modifying therapies. Accordingly, the current work is focused on the foundational step of defining reliable morphometric signatures of FTLD clinical variants relative to controls. This approach establishes the groundwork for future studies in genetically or pathologically defined cohorts. In support of this trajectory, we also include exploratory analyses of MAPT and C9orf72 carriers to illustrate how this framework may be extended toward linking morphometric signatures to genotype-confirmed pathology.

In this work, we present a novel multivariate integration and feature selection of shape morphometric features in FTLD clinical subtypes. We implemented a novel, sparse PLS method on cortical shape morphometric measures of thickness, which reflects grey matter atrophy^42^; surface curvature, which reflects sulcal widening/cortical folding,^43^; and metric distortion, which reflects white matter surface area changes and/or the shallowing of the cortical folding pattern.^44^ Given that these cortical measures are hypothesized to reflect different aspects of neuropathologic changes,^39^ we hypothesized that (1) unique signatures of cortical morphometric features would be reflected across FTLD clinical subtypes and (2) that integration of multiple morphometric features could increase the discriminatory power between the subtypes and controls in an independent, external test data set.

## Materials and methods

### Participant selection

#### ALLFTD

Data for this study were partially obtained from ALLFTD (ARTFL-LEFFTDS Longitudinal Frontotemporal Lobar Degeneration). ALLFTD represents the combination of two prior longitudinal neuroimaging studies, ARTFL and LEFFTDS, and is presently a multisite study encompassing 23 North American institutions. ALLFTD was designed to follow FTLD mutation carriers longitudinally in order to better understand the clinical and biological markers of disease progression in FTLD. Participant enrolment typically occurs due to family history suggestive of familial FTLD (i.e. prior enrolment of a symptomatic proband), however enrolment of symptomatic and asymptomatic non-carriers also occurs. Mutation carriers typically have a mutation in one of the *MAPT*, *GRN*, or *C9orf72* genes. Clinical diagnosis occurred via multidisciplinary teams employing widely accepted published criteria for each disorder,^45,46^ based on information gathered from comprehensive evaluation including neurologic assessment, caregiver or companion interview, neuropsychological testing, brain MRI, and biofluid collection. Data from individuals diagnosed with one of 3 FTLD phenotypes (bvFTD, nfvPPA, svPPA), regardless of their mutation carrier status, were included in the present analyses if they underwent a structural MRI scan at baseline. Other FTLD subtypes were not included due to insufficient sample size of symptomatic participants. Cognitively normal (CN) control participants included in the present study were asymptomatic (i.e. no detectable cognitive impairment, behavioural disturbances, or motor impairment) without a known familial mutation.

#### NIFD

Data were also obtained from the Frontotemporal Lobar Degeneration Neuroimaging Initiative (i.e. Neuroimaging in Frontotemporal Dementia, or NIFD). Participant data, including clinical and MRI measures, were collected at the University of California San Francisco, Mayo Clinic, Rochester, and Massachusetts General Hospital. Full details of subject recruitment, scanning protocols, diagnostic criteria, as well as image processing, are available at http://memory.ucsf.edu/research/studies/nifd. Diagnoses of bvFTD, nfvPPA and svPPA were made by multidisciplinary teams applying consensus diagnostic criteria (i.e. Rascovsky *et al*., 2011 for bvFTD and Gorno-Tempini *et al*., 2011 for nfvPPA/svPPA).^46,47^45omprehensive clinical evaluations for diagnostic purposes included neurologic history gathering, physical and neurologic examinations, structured caregiver interviews, neuroimaging and neuropsychological testing. Information regarding whether participants were familial mutation carriers was not included in NIFD.

#### Balanced cohort creation

To mitigate the confounding effects of unequal sample size and age distributions in our training and test groups in our sPLS-DA models, we performed a two-stage balancing and splitting procedure for each of the three pairwise comparisons (bvFTD, nfvPPA, svPPA versus CN) using pooled ALLFTD and NIFD data. First, we created a master balanced cohort for each comparison by using the smaller FTD group as a template. CN participants were then randomly subsampled to frequency-match the age distribution (stratified by quartile) and exact sample size of the corresponding FTD group. Second, each of these three master cohorts was partitioned into training (70%) and testing (30%) sets via a stratified random split on diagnosis. The entire procedure was conducted in R with a fixed random seed for reproducibility.

### Image processing

#### Structural MRI

For the ALLFTD cohort, participants were scanned at 3 Tesla (3T) on MRI scanners from one of three vendors: Philips Medical Systems, Siemens, or General Electric Medical Systems. A standard imaging protocol was used at all centres and subsequently reviewed for quality by a core group at Mayo Clinic, Rochester. The current analysis used Magnetization Prepared Rapid Gradient Echo (MPRAGE) T1-weighted images, which were acquired using the following parameters: matrix 256 × 240; about 170 slices; voxel size = 1.05 × 1.05 × 1.25 mm3; flip angle, TE and TR varied by vendor. For the NIFD cohort, 3T MRIs were acquired at one of three sites on 3T MRI scanners (T1w MPRAGE, TR = 2 ms, TE = 3 ms, IT = 900 ms, flip angle 9°, matrix 256 × 240, slice thickness 1 mm, voxel size 1mm^3^). Scan protocols between NIFD and ALLFTD were designed to ensure adequate matching between the protocols, including scanning parameters, pre-processing and image quality control.

#### FreeSurfer extraction of features

All T1-weighted MRI scans were processed through FreeSurfer version 6.0^47^ to generate cortical morphometry measures. Manual segmentation experts conducted standardized quality control procedures in accordance with FreeSurfer’s quality control guide, and any observed FreeSurfer reconstruction errors were corrected. Most errors were minor and involved slight adjustments of white matter and pial boundaries where cortical surfaces were misclassified or overextended. Next, segmented white matter volume was used to derive a tessellated surface representing the inner (grey/white matter) and outer (pial-grey) boundaries of the cortex.^48^ Cortical morphometry measures can then be estimated via the individual cortical surface.^49^ Cortical thickness (CT) was estimated as the distance between white and pial matter at each vertex, while Gaussian surface curvature (SC) was calculated as the product of the principal curvatures for each vertex on the pial surface.^50^ Jacobian metric distortion (MD) was calculated as the amount of metric distortion per vertex required to register a subject’s inflated spherical cortical surface to an average spherical surface template.^51^ Among available morphometric measures, we focused on cortical thickness, surface curvature, and metric distortion given their complementary sensitivity to cortical change, while other measures (e.g. surface area, cortical volume, gyrification, sulcal depth) were either highly collinear with chosen variables, showed minimal variability, or required additional processing beyond the scope of this study. A 15-mm FWHM kernel was applied to each cortical morphometry measure. Cortical morphometry maps were then projected onto the fsaverage non-linear average template white surface, resulting in corresponding vector sets of morphometry measures across all participants for statistical analysis and visualization. Finally, each cortical morphometry map was parcellated into 360 patches (i.e. ROIs) according to the validated MMP1 atlas from the Human Connectome Project,^52^ to aid in visualization and enhance interpretability of findings.

### Analyses

#### Group-level differences in morphometric features

All statistical comparisons were performed on the parcellated cortical surface using MATLAB 2018b^53^ and the SurfStat package (http://www.math.mcgill.ca/keith/surfstat)^54^ and visualized as a colour map on the parcellated average surface using the R package ggseg.^55^ For each morphometry measure, we conducted patch-wise linear models with diagnostic group (versus CN) as the main effect and age, sex, scanner type, and CDR-SB as covariates. This resulted in a coefficient estimate for the effect of diagnostic group, from which a t-statistic was calculated and visualized as a colour map on the overall average surface. Significance of T-scores was corrected for multiple comparisons using the Benjamini-Hochberg false discovery rate (FDR) of 0.05.

#### Sparse partial least squares discriminant analysis

To assess for the presence of multimodal imaging signatures across FTD phenotypes, we employed a data-driven integrative framework designed for high-dimensional multimodal datasets that additionally addressed the correlated nature of data types. Multimodal data integration was performed using Data Integration Analysis for Biomarker discovery using Latent cOmponents (DIABLO),^56^ which employs block sparse partial least squares-discriminant analysis (sPLS-DA). sPLS-DA operates using a supervised framework to find linear combinations of variables (e.g. neuroimaging-derived shape morphometry measures) so that the sum of covariances between each pair of data sets, including the classification outcome, is maximized. sPLS-DA was performed using the R package mixOmics.^57^ Unlike traditional PLS, which incorporates all variables into each latent component, the sparse variant applies an L1 penalty to enforce sparsity. This embedded feature selection retains only the most discriminative features, improving interpretability and reducing overfitting in high-dimensional neuroimaging data. A design matrix indicating whether data sets should be correlated is specified in sPLS-DA. To implement the optimal design matrix, individual sPLS models were run between pairwise datasets (e.g. thickness and curvature; thickness and metric distortion) which suggested that pairwise correlations across samples and groups ranged from 0.6–0.8. As such, we specified a design matrix correlation of 0.8 to reflect the upper bound of observed associations and to promote selection of features with the strongest shared structure across data types.

The optimal number of sPLS-DA components was first determined by fitting a model with up to five components and assessing global performance using leave-one-out-cross validation (LOOCV). Across tasks, the lowest balanced error rate was achieved with one component, and in a few cases with two. To maintain consistency across modalities and facilitate interpretability, final models were constrained to one component per modality. We note that allowing multiple components may capture additional variance but at the cost of interpretability and greater risk of overfitting given sample size. Following component selection, the optimal number of features (i.e. patches per shape modality) was determined via a grid search with 20 × 10–fold LOOCV. The grid of potential features was set between 7 and 35, such that the feature set selected by sPLS-DA would be of at least sufficient size to be biologically interpretable.^58^ The optimal number of features per component was defined as the grid value with the lowest classification error rate and allowed to vary per shape measure and classification task. The sPLS-DA then outputs the proportion of variance explained by each set of features per modality as well as loading vectors representing coefficients assigned to each variable, where the absolute value indicates variable importance. Loading coefficients were visualized as a colour map on the parcellated average surface using the R package ggseg.^55^

#### Model evaluation

We then evaluated the classification performance of the selected features for each comparison (i.e. bvFTD versus CN, nfvPPA versus CN, svPPA versus CN) on an independent test dataset derived from the pooled cohort and age-balanced. Classification performance was evaluated using the mixOmics predict() function. In an independent data set, mixOmics calculates a set of predicted component scores for each shape feature by using the estimated loadings vectors from the sPLS-DA. The predicted class of each new sample from the test data-set is determined using a majority vote for consensus class membership.^56^ Resulting predicted classes were input into receiver operating characteristic (ROC) curves. Additionally, we tested the predictive accuracy of the model when provided the sPLS-DA selected features from each individual shape modality separately, as well as in each possible combination with each other. This resulted in 7 possible combinations of shape features. The area under the ROC curve (AUC) was used to summarize the overall diagnostic accuracy of the model at each combination of shape features. To quantify the stability of this measure, we calculated 95% confidence intervals for each AUC using DeLong's method. Furthermore, to formally test whether combining modalities offered a significant improvement over the best single modality, we used DeLong's test for correlated ROC curves to compare the AUC of each multi-modality model against the AUC of the best-performing single-modality model. AUC values range from 0 to 1, where 1 represents a perfectly accurate model and 0.5 represents no discriminatory ability. Acceptable classification is defined as values between 0.7 and 0.8, while values over 0.8 are considered excellent.^59^

In supplemental analyses, we aimed to evaluate the selected features from sPLS-DA and their classification accuracy for the discrimination between bvFTD due to a *C9orf72* mutation (bvFTD-C9) and bvFTD due to *MAPT* mutation (bvFTD-MAPT). Familial gene carriership information was not assessed in the NIFD dataset and therefore external test data for this predictive classification could not be employed. In this case, classification performance was assessed within the original sample using 10-fold cross-validation repeated ten times. This method ran additional sPLA-DA models on multiple imputations of the sample and assesses the accuracy of the prediction on the left-out samples. Accuracy was modelled using ROC curves with associated AUC values.

## Results

### Participants

Sample demographic characteristics are presented in Table 1, stratified by cohort (i.e. ALLFTD or NIFD). Differences in study variables across diagnostic groups were assessed within each cohort using chi-square tests for categorical variables and analyses of variance (ANOVAs) for continuous variables with follow-up pairwise comparisons against CN participants where appropriate. In ALLFTD data from 173 participants were included, with 27 CN, 107 bvFTD, 27 nfvPPA, and 12 svPPA participants. Only information from the baseline MRI visit was included. The CN group was significantly younger and more likely to be female than all other diagnostic groups. As expected, the CN group was less impaired on the Montreal Cognitive Assessment (MoCA) and Clinical Dementia Rating Scale Sum of Boxes (CDR-SB) than all other diagnostic groups. Education did not significantly differ among groups. Regarding familial genotype, the bvFTD sample was comprised of 17 *C9orf72*, 5 *GRN* and 14 *MAPT* carriers, while 56 had no known mutation and genotype information for 15 participants was unavailable or unknown. The nfvPPA sample was comprised of 4 participants with *GRN* mutations, 18 without mutation and 5 were unknown. For svPPA, 1 subject had a *C9orf72* mutation, 9 were without known mutation and 2 were unknown. All CN participants used as a comparison group were negative for known mutations.

**Table 1:** Demographic characteristics in ALLFTD & NIFD cohorts

	ALLFTD					NIFD				
	bvFTD (*n* = 107)	nfvPPA (*n* = 27)	svPPA (*n* = 12)	CN (*n* = 27)	*P*	bvFTD (*n* = 66)	nfvPPA (*n* = 36)	svPPA (*n* = 29)	CN (*n* = 131)	*P*
Age	61.8 (8.1) ^a^	67.6 (6.6) ^a^	64.5 (5.5) ^a^	46.4 (12.5)	<0.001	62.2 (6.3)	68.4 (7.3)^a^	63.6 (5.9)	63.4 (7.3)	<0.001
Age Onset	56.7 (9.1)	65.0 (6.6)	60.3 (6.1)	–	*ns*	–	–	–	–	–
Sex (M/F)	60/47 a	13/14 a	7/5 a	5/22	<0.01	46/20 a	16/20	17/12	56/75	<0.01
Education	15.4 (2.5)	15.7 (2.6)	17.0 (3.2)	16.26 (2.6)	*ns*	15.6 (3.4) a	16.5 (3.4)	16.9 (3.1)	17.5 (1.9)	<0.001
MOCA	19.3 (9.9) a	23.3 (15.7) a	18.4 (8.4) a	30.52 (11.6)	<0.001	18.3 (6.4) a	21.6 (5.2) a	19.1 (4.6) a	27.5 (1.6)	<0.001
CDR-SB	5.9 (3.5) a	3.6 (3.2) a	4.2 (3.4) a	0.0 (0.0)	<0.001	5.8 (3.1) a	1.9 (2.4) a	3.7 (1.9) a	0.0 (0.2)	<0.001
Genotype (C9/GRN/MAPT/NONE/OU)	17/5/14/56/15	0/4/0/18/5	1/0/0/9/2	0/0/0/27/0	–	–	–	–	–	–

*Note.* Table showing summary of demographic variables in ALLFTD (left) and NIFD (right). Results are reported as mean (std) for continuous variables or counts for discrete variables.

^a^Indicates *P* < 0.5 for post-hoc pairwise analyses versus CN. Age and education are reported in years. MOCA = Montreal Cognitive Assessment. CDR-SB = Clinical Diagnostic Rating Scale Sum of Boxes. CN = Cognitively Normal. C9 = C9orf72. GRN = Progranulin. MAPT = Microtubule-associated protein tau. OU = Other/Unknown.

Sample characteristics of the NIFD cohort by diagnostic group are presented in Table 1 (right panel). The NIFD sample was comprised of data from 262 participants, with 131 CN, 66 bvFTD, 36 nfvPPA, and 29 svPPA. Only information from the baseline MRI visit was included. The CN group was significantly younger than the nfvPPA group and more likely to be female and more educated than the bvFTD group (all *P* < 0.01). As expected, the CN group was less impaired on the Montreal Cognitive Assessment (MoCA) and Clinical Dementia Rating Scale Sum of Boxes (CDRSB) than all other diagnostic groups. Information regarding mutation carrier status was not available for the NIFD sample.

The participants for each analysis cohort were divided into age-balanced training (70%) and testing (30%) sets. After balancing, the training set for bvFTD consisted of 119 participants with bvFTD (mean age 61.9 ± 8.0 years) and 115 controls (mean age 60.2 ± 10.6 years). The corresponding testing set included 46 participants with bvFTD (mean age 62.1 ± 6.6 years) and 43 controls (mean age 61.2 ± 10.6 years). For the nfvPPA versus CN, the training set included 44 participants with nfvPPA (mean age 68.0 ± 7.1 years) and 115 controls (mean age 60.2 ± 10.9 years). The testing set was comprised of 14 participants with nfvPPA (mean age 69.8 ± 7.0 years) and 43 controls (mean age 61.3 ± 9.7 years). Finally, for the svPPA versus control analysis, the training set was composed of 29 participants with svPPA (mean age 64.4 ± 6.2 years) and 115 controls (mean age 60.2 ± 10.9 years), while the testing set contained 10 participants with svPPA (mean age 62.5 ± 5.1 years) and 43 controls (mean age 61.1 ± 9.5 years).

Finally, sample characteristics of a smaller subset of bvFTD participants with either a *C9orf72* (bvFTD-C9) or *MAPT* (bvFTD-MAPT) mutation are included in Table 2. These groups were not significantly different with regard to age, sex, education, MoCA and CDR-SB.

**Table 2:** Demographic characteristics in bvFTD subgroups

	bvFTD-C9 (*n* = 17)	bvFTD-MAPT (*n* = 14)	*P*
Age	60.47 (10.5)	55.64 (8.2)	*ns*
Sex (M/F)	9/8	10/4	*ns*
Education	15.29 (2.5)	14.79 (2.5)	*ns*
MOCA	19.47 (6.9)	20.92 (5.8)	*ns*
CDR-SB	6.26 (4.0)	5.25 (4.6)	*ns*

*Note.* Table showing summary of demographic variables in bvFTD-C9 and bvFTD-MAPT groups. Results are reported as mean (std) for continuous variables or counts for discrete variables. Significant group differences were determined using chi-square tests for categorical variables and t-tests for continuous variables. Age and education are reported in years. MOCA = Montreal Cognitive Assessment. CDR-SB = Clinical Diagnostic Rating Scale Sum of Boxes.

### Group-level differences and discriminability of shape morphometric features

Results of group-level univariate analyses, sPLS-DA feature selection, and classification accuracy on the independent test dataset are presented for each diagnostic group below. In each corresponding figure, ‘A’ presents the results of the T-scores of patch-wise linear models for the comparison of the phenotype versus CN in the training cohort after adjustment for age, sex, CDR-SB, scanner type and additionally adjusted for multiple comparisons correction (i.e. T-scores where *P*_FDR_ < 0.05). B presents the loading coefficients of the sPLS-DA feature selection for the discrimination task and C presents the results of combinations of the sPLS-DA-selected features when in entered into a classification task either using independent, external data or via cross-validation.

### bvFTD versus CN

**Figure 1:**
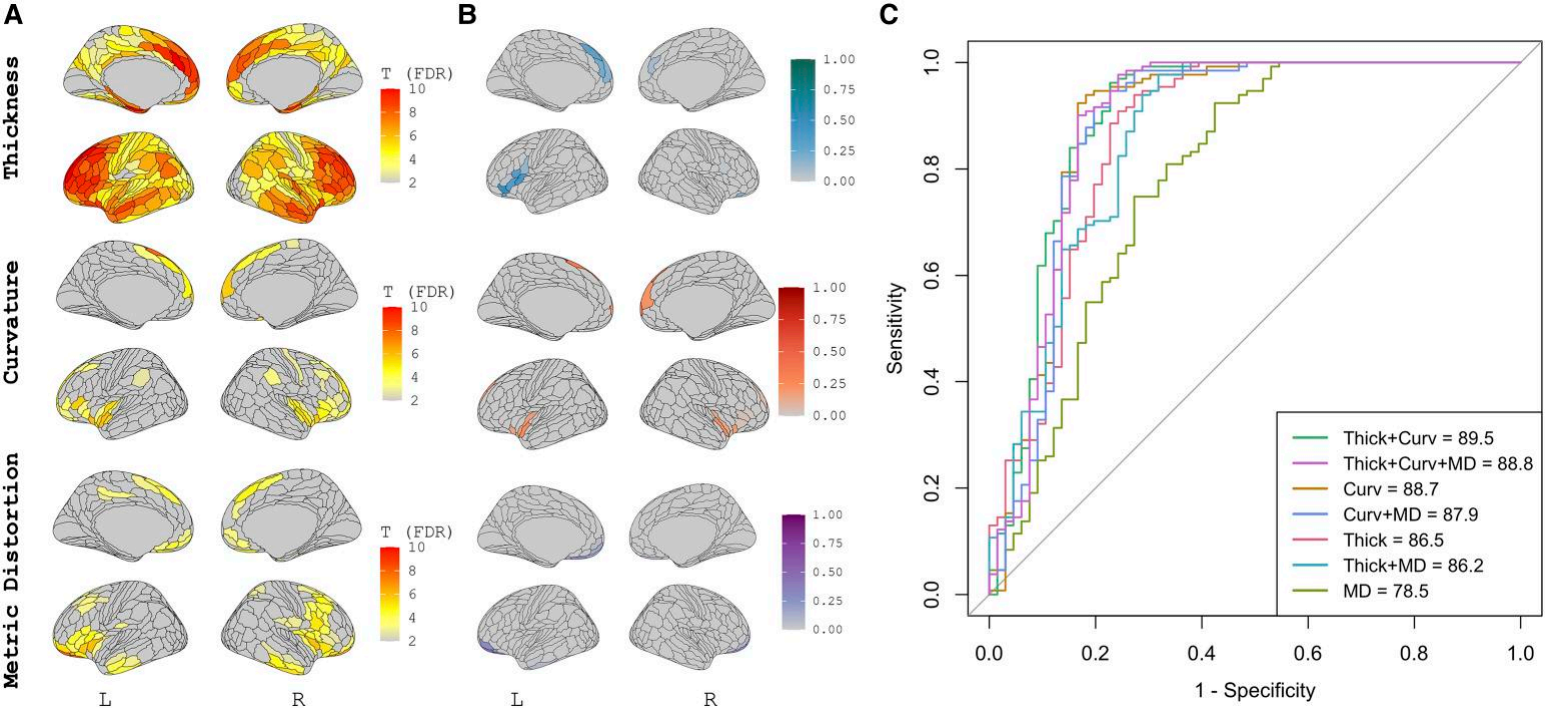
**Univariate and multivariate analysis of morphometric features in bvFTD.** Training (*n* = 119 bvFTD, *n* = 115 CN), Testing (*n* = 46 bvFTD, *n* = 43 CN). Results of the univariate analyses **(A)**, sPLS-DA feature selection **(B)**, and classification accuracy **(C)** for bvFTD versus CN. In A & B, results derived from cortical thickness are presented in the top panel, surface curvature in the middle panel and metric distortion in the bottom panel. A represents T-scores of patch-wise linear models for the comparison of bvFTD versus CN after adjustment for age, sex, scanner, and CDR-SB and additionally adjusted for multiple comparisons correction (*P*_FDR_ < 0.05). B presents the loading coefficients of the sPLS-DA feature selection for the discrimination task of bvFTD versus CN. Absolute values are presented at various colours scales and represent the contribution of the patch to the discrimination task. C presents the results of features in B entered into a classification task using independent data. AUC = area under the curve. MD = metric distortion. Thick = cortical thickness. Curv = surface curvature. sPLS-DA = sparse partial least squares discriminant analysis. bvFTD = behavioural variant frontotemporal dementia. CN = cognitively normal. FDR = false discovery rate.

Results of the univariate analyses, sPLS-DA feature selection, and classification accuracy for bvFTD versus CN are presented in Fig. 1. bvFTD participants showed robust cortical atrophy (i.e. low cortical thickness) throughout the bilateral inferior frontal/insular cortex, anterior temporal lobe and anterior cingulate (Fig. 1A, left). Surface curvature abnormalities were noted in the bilateral anterior cingulate/medial prefrontal region and the bilateral insula (Fig. 1A, middle). Significantly discrepant metric distortion was observed in the bilateral orbitofrontal cortex and the anterior and middle temporal cortex (Fig. 1A, right). Results of the sPLS-DA revealed that the selected multimodal signature for discrimination between bvFTD and CN explained 29.8% of the variance in cortical thickness, 12.2% of the variance in surface curvature, and 6.3% of the variance in metric distortion. Analysis of the loading coefficients indicated a multimodal signature involving cortical thickness in the left inferior frontal/insular and anterior cingulate cortex, alongside bilateral anterior cingulate and insular surface curvature and orbitofrontal metric distortion, best discriminated between bvFTD and CN. When assessed on the independent test data set, the features from cortical thickness and surface curvature combined produced the highest AUC (89.5, 95% CI = 83.0–95.4), followed by all three measures combined (AUC = 88.8, 95% CI = 82.0–94.7) and curvature alone (AUC = 88.7, 95% CI = 82.1–95.1). The combination of cortical thickness and curvature was significantly greater than the highest-performing single measure (curvature), *P* = 0.04; no other significant differences between combined metrics and curvature were noted.

### nfvPPA versus CN

**Figure 2:**
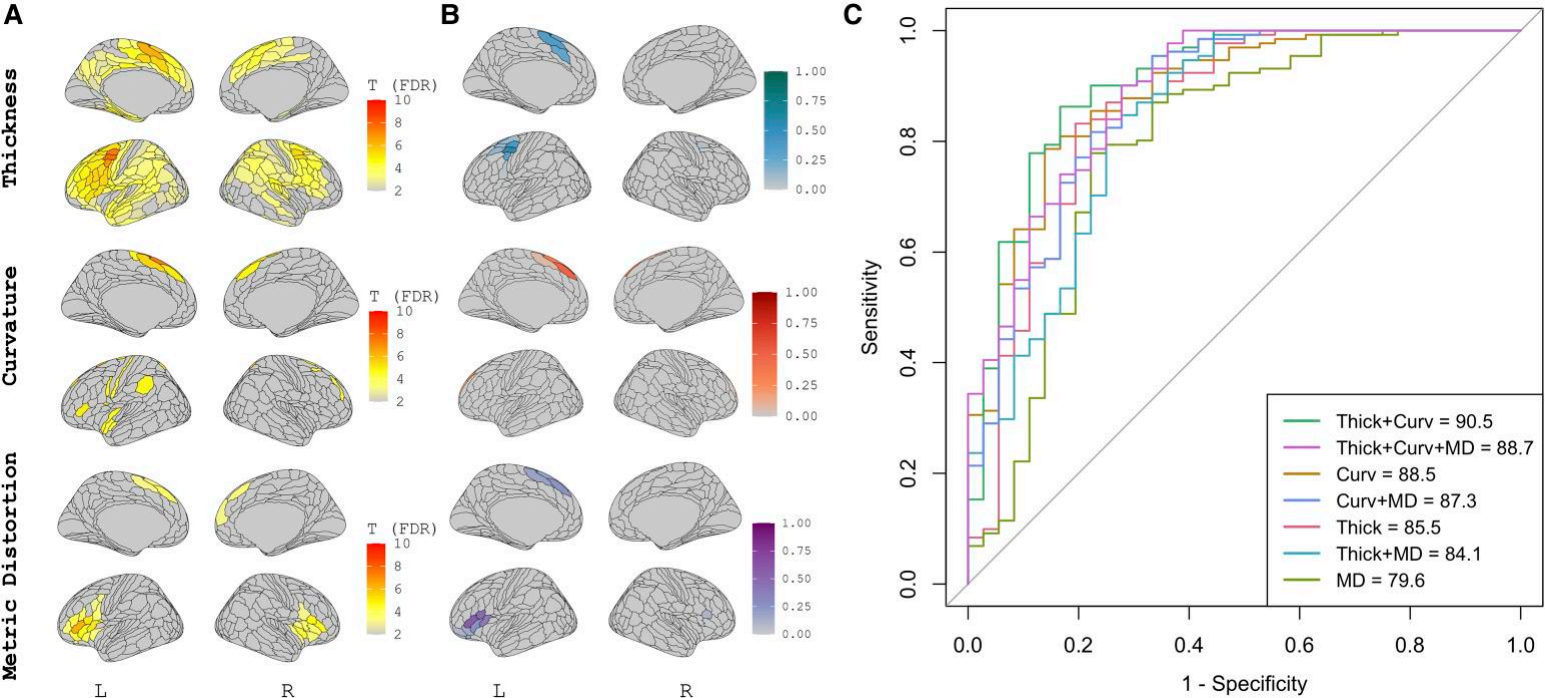
**Univariate and multivariate analysis of morphometric features in nfvPPA.** Training (*n* = 44 nfvPPA, *n* = 115 CN), Testing (*n* = 14 nfvPPA, *n* = 43 CN). Results of the univariate analyses **(A)**, sPLS-DA feature selection **(B)**, and classification accuracy **(C)** for nfvPPA versus CN. In A & B, results derived from cortical thickness are presented in the top panel, surface curvature in the middle panel and metric distortion in the bottom panel. A represents T-scores of patch-wise linear models for the comparison of bvFTD versus CN after adjustment for age, sex, scanner, and CDR-SB and additionally adjusted for multiple comparisons correction (*P*_FDR_ < 0.05). B presents the loading coefficients of the sPLS-DA feature selection for the discrimination task of nfvPPA versus CN. Absolute values are presented at various colours scales and represent the contribution of the patch to the discrimination task. C presents the results of features in B entered into a classification task using independent data. AUC = area under the curve. MD = metric distortion. Thick = cortical thickness. Curv = surface curvature. sPLS-DA = sparse partial least squares discriminant analysis. CN = cognitively normal. nfvPPA = non-fluent variant primary progressive aphasia. FDR = false discovery rate.

Results of the univariate analyses, sPLS-DA feature selection, and classification accuracy for nfvPPA versus CN are presented in Fig. 2. nfvPPA participants showed cortical atrophy in the bilateral (left greater than right) inferior prefrontal, premotor and fronto-opercular, and para/midcingulate cortex (Fig. 1A, left). Alterations in surface curvature were noted in the bilateral (left greater than right) anterior and midcingulate, and somatomotor cortex (Fig. 1A, middle). Metric distortion abnormalities were observed primarily in the left inferior cortex, Brodmann areas 44/45 (Fig. 1A, right). Results of the sPLS-DA revealed that the selected multimodal signature for discrimination between nfvPPA and CN explained 31.9% of the variance in cortical thickness, 11.5% of the variance in surface curvature, and 7.7% of the variance in metric distortion. Analysis of the loading coefficients indicated a multimodal signature involving cortical thickness of the left premotor/midcingulate, surface curvature in the left anterior/midcingulate cortex and metric distortion in the left inferior frontal and orbitofrontal cortex best discriminated between nfvPPA and CN. When assessed on the independent test data set, the features from cortical thickness and surface curvature produced an outstanding AUC of 90.5 (95% CI = 83.5–96.9), followed by all three measures combined (AUC = 88.7, 95% CI = 82.1–95.1) and curvature alone (AUC = 88.5, 95% CI = 81.6–94.6). The combination of cortical thickness and curvature was not statistically greater than the highest-performing single measure (curvature), *P* = 0.13, though was statistically higher than cortical thickness alone, *P* < 0.01.

### svPPA versus CN

**Figure 3:**
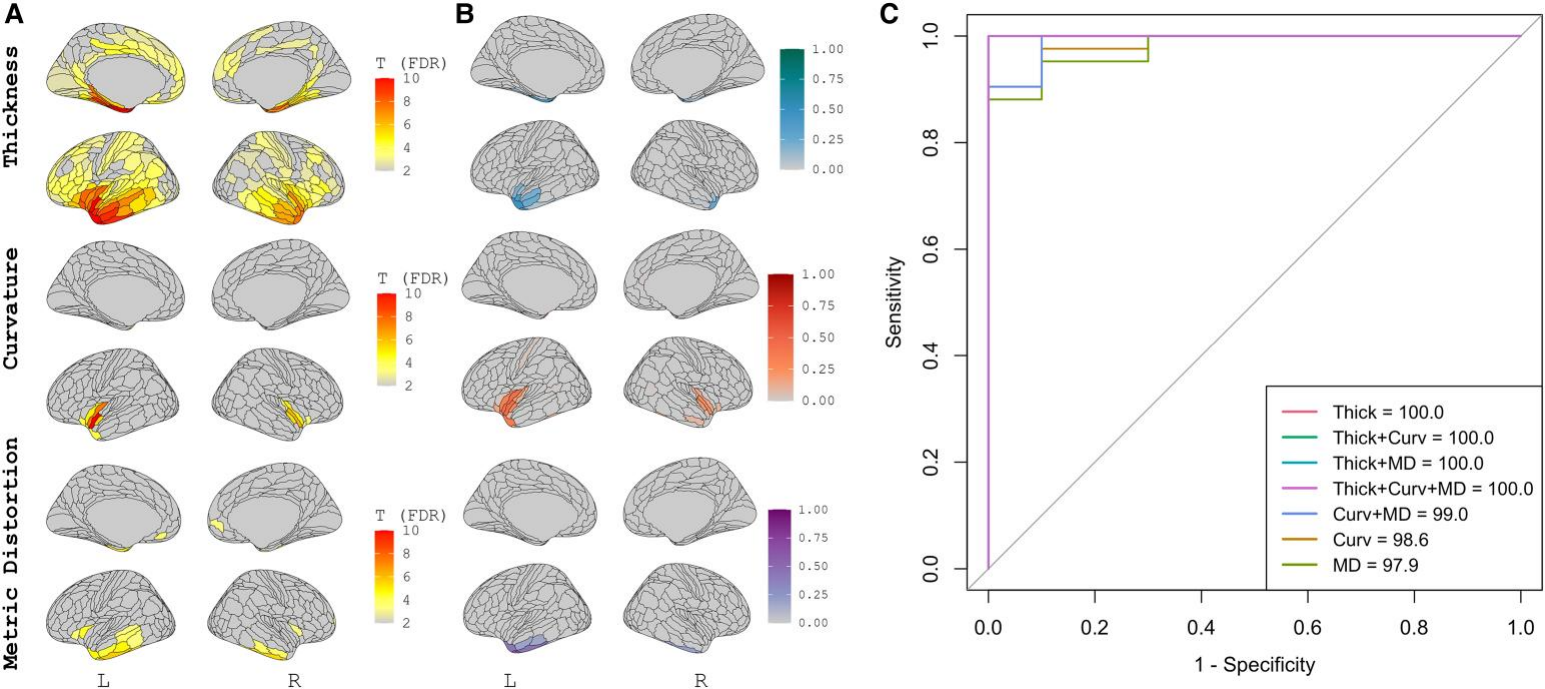
**Univariate and multivariate analysis of morphometric features in svPPA.** Training (*n* = 29 svPPA, *n* = 115 CN), Testing (*n* = 10 svPPA, *n* = 43 CN). Results of the univariate analyses **(A)**, sPLS-DA feature selection **(B)**, and classification accuracy **(C)** for svPPA versus CN. In A & B, results derived from cortical thickness are presented in the top panel, surface curvature in the middle panel and metric distortion in the bottom panel. A represents T-scores of patch-wise linear models for the comparison of bvFTD versus CN after adjustment for age, sex, scanner, and CDR-SB and additionally adjusted for multiple comparisons correction (*P*_FDR_ < 0.05). B presents the loading coefficients of the sPLS-DA feature selection for the discrimination task of svPPA versus CN. Absolute values are presented at various colours scales and represent the contribution of the patch to the discrimination task. C presents the results of features in B entered into a classification task using independent data. AUC = area under the curve. MD = metric distortion. Thick = cortical thickness. Curv = surface curvature. sPLS-DA = sparse partial least squares discriminant analysis. CN = cognitively normal. svPPA = semantic variant primary progressive aphasia. FDR = false discovery rate.

Figure 3 presents the results of the univariate analyses, sPLS-DA feature selection, and classification accuracy for svPPA versus CN. svPPA participants showed a canonical pattern of striking asymmetric (left greater than right) anterior temporal lobe atrophy (Fig. 1A, left). Additional cortical thickness was noted in left insular and medial temporal cortex. Surface curvature abnormalities were noted in the bilateral (left greater than right) anterior temporal and insular cortex (Fig. 1A, middle). Significantly discrepant metric distortion was observed in the bilateral temporal lobes and anterior cingulate, with highest T-scores in the left superior temporal gyrus (Fig. 1A, right). Results of the sPLS-DA revealed that the selected multimodal signature for discrimination between svPPA and CN explained 22.4% of the variance in cortical thickness, 6.5% of the variance in surface curvature, and 7.9% of the variance in metric distortion. Analysis of the loading coefficients indicated a multimodal signature involving cortical thickness and surface curvature in the left insular and anterior cingulate cortex alongside left lateral inferior and superior temporal metric distortion best discriminated between svPPA and CN. When assessed on the independent test data set, any set of features including cortical thickness maximized model performance at AUC of 100.

### bvFTD-C9 versus bvFTD-MAPT

**Figure 4:**
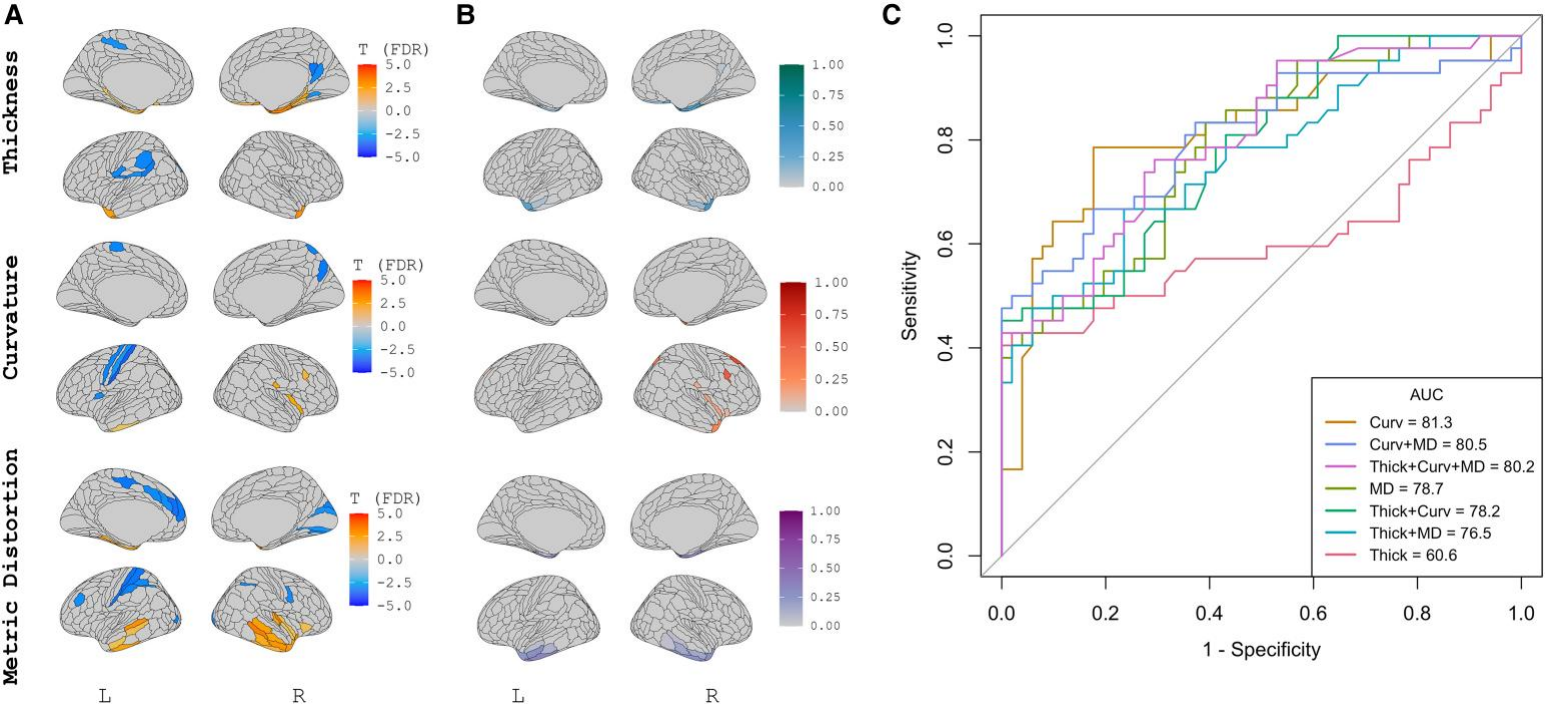
**Univariate and multivariate analysis of morphometric features in bvFTD-MAPT (*N* = 14) and bvFTD-C9 (*N* = 17).** Results of the univariate analyses **(A)**, sPLS-DA feature selection **(B)**, and classification accuracy **(C)** for bvFTD-C9 versus bvFTD-MAPT. In A & B, results derived from cortical thickness are presented in the top panel, surface curvature in the middle panel and metric distortion in the bottom panel. A represents T-scores of patch-wise linear models for the comparison of bvFTD-C9 versus bvFTD-MAPT after adjustment for age, sex, scanner, and CDR-SB and additionally adjusted for multiple comparisons correction (*P*_FDR_ < 0.05). Areas in red (positive T) and blue (negative T) represent aberrant/pathological effects in bvFTD-MAPT and bvFTD-C9, respectively. B presents the loading coefficients of the sPLS-DA feature selection for the discrimination task of bvFTD-C9 versus bvFTD-MAPT. Absolute values are presented at various colours scales and represent the contribution of the patch to the discrimination task. C presents the results of features in B entered into a classification task using leave one out cross-validation. AUC = area under the curve. MD = metric distortion. Thick = cortical thickness. Curv = surface curvature. sPLS-DA = sparse partial least squares discriminant analysis. bvFTD = behavioural variant frontotemporal dementia. MAPT = microtubule-associated protein tau. C9 = chromosome 9 open reading frame 72. FDR = false discovery rate.

Finally, we conducted complementary analyses on a subsample of 17 bvFTD-C9 and 14 bvFTD-MAPT participants. Univariate analyses were conducted comparing cortical thickness, surface curvature, metric distortion values between the two groups after adjusting for age, sex, CDR-SB, and scanner type. Univariate analyses versus non-carrier control participants are included in Supplemental Fig. 1. In Fig. 4A, areas in coloured in orange/red represent significant (FDR-corrected) T-scores where bvFTD-MAPT display aberrant cortical thickness, surface curvature or metric distortion as compared to bvFTD-C9, whereas areas in blue display aberrant cortical thickness, surface curvature or metric distortion in bvFTD-C9 as compared to bvFTD-MAPT. We observed increased cortical atrophy in bvFTD-MAPT in the bilateral anterior and medial temporal lobes, while bvFTD-C9 showed greater cortical atrophy in the left inferior parietal cortex and right posterior cingulate. Aberrant surface curvature was observed in the right posterior insula in bvFTD-MAPT and in the left somatomotor and right posterior cingulate in bvFTD-C9. Aberrant metric distortion of the bilateral (right greater than left) lateral inferior and superior temporal cortex was observed in bvFTD-MAPT, while in bvFTD-C9 there was evidence of abnormal metric distortion in the left somatomotor, inferior parietal and anterior cingulate cortex. Results of the sPLS-DA revealed that the selected multimodal signature for discrimination between bvFTD-C9 and bvFTD-MAPT explained 23.7% of the variance in cortical thickness, 11.0% of the variance in surface curvature, and 9.8% of the variance in metric distortion. Analysis of the loading coefficients indicated a multimodal signature involving cortical thickness in the bilateral medial temporal lobe, surface curvature in the right anterior temporal/insular cortex and metric distortion in the lateral inferior temporal cortex best discriminated between bvFTD-C9 and bvFTD-MAPT. Classification accuracy as assessed by LOOCV indicated measures of surface curvature alone provided the best classification accuracy at AUC = 81.3 (95% CI = 75.1–87.5), followed by the combination of surface curvature and metric distortion (AUC = 80.5, 95% CI = 74.2–86.8) and all three measures combined (AUC = 80.2, 95% CI = 73.9–86.5). Notably, all models that included surface curvature significantly outperformed the model based on cortical thickness alone (*P* < 0.05 for all comparisons).

## Discussion

In the present study, we present findings from a novel, multivariate integration of cortical morphometric features among the FTLD clinical phenotypes of bvFTD, nfvPPA and svPPA. Our approach both integrated and selected discriminative features from multiple modalities of morphometric information, thus aiding in the creation of a multimodal morphometric signature of each clinical phenotype. Finally, we demonstrate that in select classification tasks, the integrated features showed improvement above typical cortical thickness measures in the diagnostic power of MRI measures to discriminate between phenotypes and controls among an independent test dataset. The results provide support for the utility of multivariate integration methods for the identification of meaningful neurobiological markers of FTLD clinical subtypes.

In bvFTD, we observed a pattern of significant cortical atrophy as compared to controls across the frontotemporal regions of the brain, concentrated in the bilateral inferior frontal/insula and anterior cingulate. This is consistent with prior investigations of unimodal volumetric approaches measuring atrophy in this group,^18^ as well as with the hypothesized neuropathologic origin of bvFTD among selectively vulnerable fork cells and von Economo (VE) neurons in these areas.^60^ Further, we observed alterations in surface curvature in the bilateral anterior cingulate and metric distortion of the orbitofrontal cortex. While we are unaware of extant literature examining surface curvature or metric distortion in bvFTD, these measures have been hypothesized to reflect sulcal widening and white matter surface area, respectively.^42,43^ In bvFTD, white matter abnormalities have been noted among many major fibre tracts of the brain, although typically involving connections within or between frontal and temporal cortices, such as the superior and inferior longitudinal fasciculi, anterior cingulum, corpus callosum, uncinate fasciculus, fronto-striatal and fronto-thalamic pathways.^61-64^ Interestingly, while areas of greatest surface curvature and metric distortion abnormality corresponded with regions showing evidence of cortical atrophy, they were not entirely concordant with atrophy patterns, nor with each other. It is possible that these differences reflect susceptibility of those cortical regions to distortions of surface curvature or metric distortion, as alterations of cingulate curvature have been observed in schizophrenia^65^ and normal aging.^66^ Alternatively, they may reflect unique aspects of neurodegeneration, such that surface curvature alterations may reflect sulcal widening specific to effects of FTLD pathology in the area, possibly as a result of selective loss of VE neurons of the anterior cingulate.^67^ In that vein, metric distortion of the orbitofrontal cortex may reflect metabolic connectivity disturbances that have been observed to selectively impact this region in bvFTD.^68^ However, future work correlating the observed metric distortion and surface curvature abnormalities with neuropathological or connectivity-based modalities is necessary in order to fully understand these effects.

Results of the sPLS-DA analysing discriminability of shape morphometric features between bvFTD and CN revealed a multimodal signature comprised of cortical thickness in the left inferior frontal and anterior cingulate, surface curvature of the anterior cingulate and metric distortion in the orbitofrontal cortex. In general, regions identified for each morphometric feature corresponded to an area identified as abnormal as compared to controls in univariate analyses. Notably, the features selected by the sPLS-DA included cortical thickness and metric distortion of the left hemisphere alongside surface curvature of the right hemisphere. Studies of neurodegeneration in bvFTD have typically implicated the right hemisphere more than the left based on structural MRI.^69,70^ However, hemispheric discrepancies have been hypothesized to be the result of heterogeneity in underlying neuropathology, as atrophy of the right orbitofrontal cortex was shown to be more common in bvFTD due to FTLD-tau and atrophy of the left ventrolateral temporal lobes was observed in those with TDP-43 pathology.^71^ When the multimodal signature was entered iteratively into a classification task on independent test data, we determined that a combined signature including cortical thickness and surface curvature provided the best AUC. Of measures assessed independently, surface curvature slightly outperformed cortical thickness and metric distortion. Contrastingly, the sPLS-DA-derived multimodal signature explained a substantially greater proportion of the variance of cortical thickness than surface curvature. These results suggest that while cortical thickness is most sensitive to bvFTD-related change, surface curvature is more specific. However, a more in-depth examination of the manner in which shape morphometric features degrade over time will be necessary to fully disentangle these effects. Indeed, it is possible that surface curvature changes occur only late in the disease process and therefore are more sensitive to bvFTD diagnosis, whereas cortical thickness changes are common among normal aging and/or individuals with an incipient disease process and therefore are less specific. Taken together, these findings suggest that cortical thickness most directly reflects neuronal loss and atrophy, surface curvature may capture sulcal widening linked to selective neuronal vulnerability, and metric distortion reflects local area expansion/contraction. The comparatively smaller contribution of metric distortion in our models may indicate that areal changes are subtler than thinning or folding abnormalities in FTLD, though orbitofrontal abnormalities highlight its potential sensitivity to connectivity-related disturbances.

Primary progressive aphasia (PPA) is a clinical syndrome caused by neurodegeneration of the language-dominant hemisphere, resulting in impaired language functions early in the disease course.^72^ PPA can be subdivided into variants that tend to associate with aggregation of specific pathologic proteins in select regions of the brain. nfvPPA is primarily defined by language dysfunction involving disrupted sentence structure, abnormal syntax, and misuse of word-endings (i.e. grammatical morphemes), but also includes individuals with apraxia of speech without clear-cut language deficits.^73^ In our univariate analyses examining nfvPPA versus controls, we found cortical atrophy in the bilateral (left greater than right) inferior prefrontal, premotor and para/midcingulate cortex, consistent with prior research.^74^ We additionally found surface curvature abnormalities of the bilateral (left greater than right) anterior and midcingulate and somatomotor cortex, as well as metric distortion abnormalities, which were observed primarily in the left inferior frontal cortex corresponding with Brodmann areas 44/45. Results of the sPLS-DA analysing discriminability of morphometric features between nfvPPA and CN revealed a multimodal signature comprised of bilateral inferior frontal/insular and anterior cingulate cortical thickness, anterior cingulate surface curvature and metric distortion in the left inferior frontal/insular cortex. When these regions were assessed within a classification task on an independent test data set, we determined that a combined signature including cortical thickness and surface curvature provided the best AUC. Qualitatively, results of both our univariate and sPLS-DA analyses in nfvPPA compared to CN were similar to those in bvFTD, with a few notable distinctions. In the sPLS-DA, regions of cortical thickness contributing to the discrimination between nfvPPA and CN were predominantly inferior frontal, whereas those distinguishing bvFTD were more dorsolateral prefrontal. Moreover, regions of metric distortion that contributed to the discrimination between nfvPPA were more concentrated in the left inferior frontal/insular regions as compared to the orbitofrontal cortex in bvFTD.

Similarities in the discriminative cortical morphometric features between bvFTD and nfvPPA are not surprising, given both are frequently underlain by with FTLD-Tau pathology,^72,75^ our work corroborates that of others who have indicated there are some key differences between the neurodegeneration patterns of nfvPPA and bvFTD. Indeed, Mandelli and colleagues^76^ found that nfvPPA participants showed greater atrophy in the left posterior insula, which corresponds more to speech-production, whereas bvFTD participants showed greater atrophy in the ventral anterior insula, which corresponds to social-emotional functions. We observed similar results in our sPLS-DA; however, the discrepancy was most apparent within metric distortion in the posterior insula/Broca’s area in nfvPPA as compared to metric distortion in the orbitofrontal cortex for bvFTD. Our work highlights that metric distortion may provide incremental, though significant, value to discriminate between nfvPPA and bvFTD; however, this should be explored further in future research.

The semantic variant of PPA is primarily defined by impaired naming due to single-word comprehension deficits.^77^ Pathological analysis of post-mortem brain tissue in svPPA has revealed it is most commonly associated with FTLD-TDP-43 Type C pathology.^72,78^ As was seen in our univariate analyses, svPPA is typically associated with atrophy of the left temporal pole, a brain region known to subserve language functions including naming.^19^ Interestingly, evaluation of surface curvature in our sample revealed a similar, yet considerably milder, pattern of abnormality slightly favouring the insula/opercular regions. However, metric distortion abnormalities were observed in the lateral inferior temporal cortex. Longitudinal studies of atrophy in svPPA indicate a pattern of atrophy that spreads over time posteriorly along the temporal lobe.^74^ Future longitudinal work should examine whether this metric distortion abnormality is indicative of longitudinal progression of neurodegeneration beyond the temporal pole, or if it is reflective of methodological limitations of the metric distortion measure (i.e. metric distortion is not sensitive to neurodegeneration of the temporal pole). In our sPLS-DA and independent test classification analyses, we observed that cortical thickness measures perfectly discriminated svPPA from CN among independent test data. This finding is consistent with prior work indicating that the distinctive effects of TDP-43 Type C results in a unique pattern of neurodegeneration among FTLD syndromes^79,80^ and is thus easily distinguished.

Finally, we conducted complementary analyses among two familial mutation carrier subgroups of bvFTD, bvFTD-C9 and bvFTD-MAPT. In univariate analyses, we found small but significant differences between bvFTD-C9 and bvFTD-MAPT across morphometric measures of cortical thickness, surface curvature, and metric distortion. Generally, abnormalities in bvFTD-MAPT tended to be more concentrated in the medial temporal lobe for cortical thickness, and the lateral temporal lobe for metric distortion. bvFTD-C9 showed more abnormality in the inferior parietal for cortical thickness, the somatomotor cortex for surface curvature and the anterior cingulate and inferior parietal for metric distortion. Interestingly, the sPLS-DA selected regions that were more abnormal in bvFTD-MAPT compared to bvFTD-C9 as those that best discriminate between the two groups. Classification accuracy as assessed by the AUC of cross-validation analyses indicated excellent AUC for measures of surface curvature alone at 81.3, closely followed by surface curvature and metric distortion combined at 80.5. The pattern of surface curvature that was associated with the superior discrimination in cross-validation analyses included abnormalities in the right anterior temporal and insular cortex, in areas where bvFTD-MAPT was more impaired. In studies of presymptomatic *MAPT* mutation carriers, the medial and temporal lobes are consistently implicated.^81-85^ Similarly, the insular and supplementary motor areas, which contributed to the discrimination of bvFTD-MAPT from bvFTD-C9 in our analysis, have previously been implicated in the presymptomatic stage of MAPT-related bvFTD.^85^

The observed morphometric differences likely reflect underlying molecular and genetic differences. Mutations in the MAPT gene result in tauopathy, with pathological inclusions composed of hyperphosphorylated tau. The specific form of tauopathy can vary depending on the mutation and may involve predominantly 3-repeat (3R), 4-repeat (4R), or mixed 3R + 4R tau isoforms.^86^ These tau inclusions disrupt microtubule stability and axonal transport, often leading to early and focal degeneration in medial temporal regions. In contrast, expansions in the C9orf72 gene typically lead to the accumulation of TDP-43 type B pathology, seen in both FTD and ALS.^87^ This distinct pathological cascade is associated with more diffuse cortical involvement, though in clinical presentations of bvFTD, typically in the anterior cingulate and orbitofrontal regions.^88^ Thus, the sensitivity to detect differences between clinically similar disease presentations allows for the potential determination of underlying neuropathological processes, which is essential for treatment considerations in the advent of disease modifying therapies.

While some have suggested that different mutations producing a bvFTD syndrome may ultimately converge on similar patterns of neurodegeneration, our results suggest that subtle morphometric features may still allow for differentiation between MAPT and C9orf72 carriers.^89^ Ultimately, comparisons across both presymptomatic and symptomatic mutation carriers, especially those with varying clinical phenotypes, will be essential to clarify the biological mechanisms that drive the morphometric differences identified in the present study. Although large datasets like ALLFTD include presymptomatic mutation carriers, many individuals have not yet converted, limiting the ability to conduct this type of direct group-wise morphometric analysis. Future longitudinal studies with confirmed converters will be critical to mapping the progression of structural changes over time.

This study has notable limitations. First, we were limited by sample size, particularly within the mutation carrier subgroups, which constrained our ability to perform gene-specific analyses across the full spectrum of genetic FTD. Second, we focused only on three clinical phenotypes of FTLD, and the extent to which our findings generalize to other subtypes remains unknown. Third, the bvFTD-C9 versus bvFTD-MAPT classification analysis relied on cross-validation rather than an independent test set, which may inflate classification performance estimates and limits the generalizability of these results. We also examined only three surface-based morphometric features – cortical thickness, surface curvature, and metric distortion – selected to balance complementary information with parsimony. However, we acknowledge that additional morphometric measures may provide further insights not available in the current work. Both curvature and sulcal depth reflect folding patterns; however in this work curvature was prioritized as it has been proposed to reflect local convexity/concavity of the cortex, including from secondary/tertiary folds, as opposed to the more primary linear displacement quantified by sulcal depth.^51,90,91^ Similarly, surface area was not included in this study to reduce input dimensionality, as metric distortion has been shown to correlate with area^92^ due to shared features of surface expansion.^93^ Additional features such as subcortical volume, cerebellum/brainstem, or functional connectivity could further enhance discriminatory power. Finally, although metric distortion contributed less prominently than thickness or curvature to classification, it may capture subtle aspects of disease progression or connectivity-related vulnerability not reflected in other measures and should be examined in larger or longitudinal datasets. The multivariate modelling approach used here also has inherent limitations. Sparse PLS-DA requires parameter tuning for component number and feature selection thresholds, and performance estimates can be sensitive to these choices, particularly in modest sample sizes relative to feature dimensionality. Moreover, while sPLS-DA improves interpretability by enforcing sparsity, the resulting components represent latent multivariate patterns rather than direct regional effects, which can complicate biological interpretation. Future work using complementary multivariate frameworks, larger samples, and replication across cohorts will be essential to confirm the stability and generalizability of the identified morphometric signatures. Nonetheless, this work represents a relatively rare application of a machine learning-based approach in FTLD, systematically assessing the relative contributions of multiple morphometric features across a large, multi-site dataset. Our integration of univariate and multivariate analyses allowed us to highlight both localized group differences and broader patterns that contribute to classification performance, laying important groundwork for future replication and expansion in larger, genetically stratified cohorts.

In summary, we present here a unique study evaluating shape morphometric features among FTLD clinical phenotypes. Further, we employed novel multivariate methods to allow for feature selection of morphometric features that best discriminate between phenotypes and controls. Overall, we found that integrating surface morphometric features, in particular surface curvature and cortical thickness, improved classification performance for bvFTD and nfvPPA versus controls. Moreover, the sPLS-DA approach indicated that distinctive brain regions contribute to discrimination for each morphometric feature, suggesting they may reflect unique aspects of neurodegeneration across groups. We believe this method could prove invaluable in future studies for the early detection of FTLD phenotypes. However, this work is just the first step of many in understanding how morphometric features degrade across time, across FTLD phenotypes, and across genetic mutation carrier groups, and further longitudinal studies are necessary to optimally disentangle the causes of the unique patterns of neurodegeneration observed in the present study.

## Supplementary Material

fcag012_Supplementary_Data

## Data Availability

The data utilized in this research is sourced from the ALLFTD Consortium. Access to ALLFTD Consortium data is subject to their established data sharing policies and agreements. Researchers seeking access to the data should refer to the ALLFTD Consortium's official website or contact their data management and access committee. Detailed information on data availability, access procedures, and any potential usage restrictions can be obtained from the Consortium's official channels.
