# Supplementary material for: Morphometric features enhance phenotype discrimination in frontotemporal lobar degeneration
Source: Brain Commun. 2026 Feb 2;8(1):fcag012. doi: 10.1093/braincomms/fcag012 (PMC12887737; doi:10.1093/braincomms/fcag012)
Supplement: fcag012_Supplementary_Data [file fcag012_supplementary_data.docx]

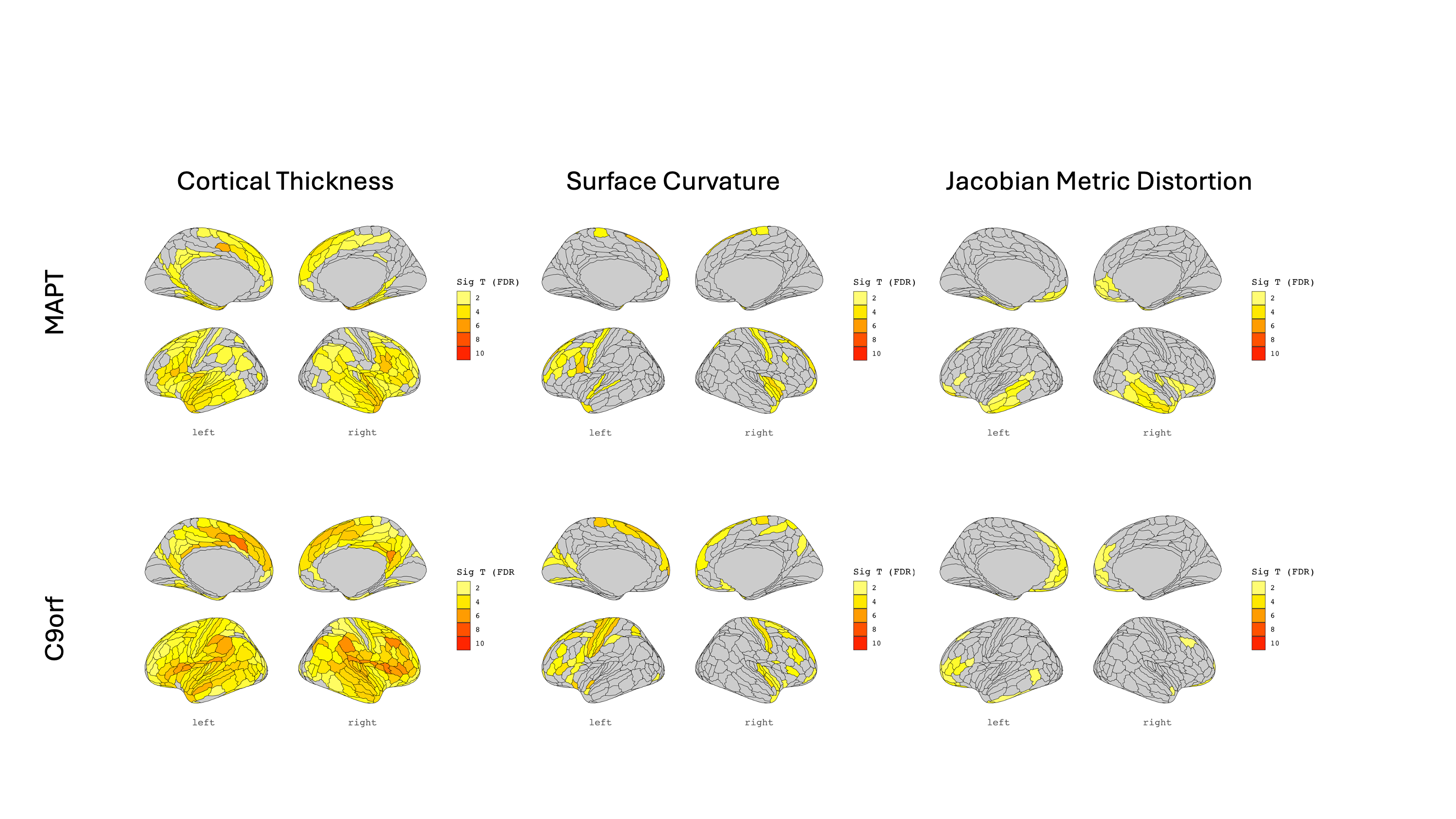


**Supplemental Figure 1.** Results of the univariate analyses assessing T-scores of patch-wise linear models for the comparison of bvFTD-MAPT (top, N = 14) and bvFTD-C9 (bottom, N = 19) vs controls after adjustment for age, sex, education, and scanner and additionally adjusted for multiple comparisons correction (p_FDR_<.05). Areas in yellow-red (positive T) represent aberrant/pathological effects in patient group.
